# The functional indexes of RBCs and microcirculation in the traumatic brain injury with the action of 2-ethil-6-methil-3-hydroxypiridin succinate

**DOI:** 10.1186/s12868-021-00657-w

**Published:** 2021-09-15

**Authors:** V. Polozova Anastasia, A. Boyarinov Gennadii, O. Nikolsky Viktor, V. Zolotova Marina, V. Deryugina Anna

**Affiliations:** 1 Department of Physiology and Anatomy, Institute of Biology and Biomedicine, Lobachevsky University, 23 Gagarin Ave., Nizhny Novgorod, Russia; 2 Department of Anesthesiology and Intensive Care, Privolzhsky Research Medical University of the Ministry of Health of the Russian Federation, Nizhny Novgorod, Russia

**Keywords:** Traumatic brain injury (TBI), RBC (red blood cells), Microcirculation, 2-ethil-6-methil-3-hydroxipiridin succinate

## Abstract

**Research aim:**

To study the RBCs functional and metabolic parameters and the microcirculatory brain structure at traumatic brain injury (TBI) under the action of 2-ethyl-6-methyl-3-hydroxypyridine succinate.

**Methods:**

A closed TBI was modeled by the free fall of a load on the parietooccipital regions of head. We made studies of the influence of 2-ethil-6-methil-3-hydroxipiridin succinate on aggregation and electrophoretic mobility of RBCs, catalase activity, malonic dialdehyde concentration, adenosine triphosphate and 2.3-biphosphoglycerate (2.3 – BPG) concentrations in RBCs. The state of parenchyma and microcirculatory brain mainstream in post-traumatic period of TBI have been studied on micro-preparations.

**Results:**

The use of 2-ethyl-6-methyl-3-hydroxypyridine succinate under conditions of head injury leads to a decrease in MDA concentration and in aggregation of RBCs, to an increase in the 2.3—BPG concentration and RBC electrophoretic mobility compared to the control (group value). The most pronounced changes under the action of 2-ethyl-6-methyl-3-hydroxypyridine succinate were observed 3–7 days after the TBI. Significant indicators of the restoration of the microvasculature and brain tissue provoked by the use of 2-ethyl-6-methyl-3-hydroxypyridine succinate of were evident from the 7th day unlike the control group, where the restoration of structural morphological parameters was observed only on the 12th day of the post-traumatic period. Fast recovery of blood flow under the action of 2-ethyl-6-methyl-3-hydroxypyridine succinate ensured effective restoration of neurons and glia in comparison with the control group.

**Conclusions:**

Early and long-term cytoprotective correction intensifies the oxygen transport function of the blood, prevents and / or reduces disorders of microvessels, neurons and glia in the post-traumatic period, thereby provides correction of hypoxic state and drives to the restoration of brain tissues homeostasis.

## Background

Traumatic brain injury (TBI) is the most difficult multidisciplinary, medical, biological, and social problem due to a persistent high prevalence of death and disablement of patients of the most active working age [1–4]. In multiple researches, devoted to TBI problem, the role of secondary mechanisms of brain trauma is shown. Their trigging provokes the broadening of primary lesion foci and formation of new pathological processes abruptly making worse the clinical course and trauma outcome [5, 6].

In post-traumatic period, the quickest interrelated changes with oxygen status, hemorheology, acid—basis and water-electrolytic states take place [7]. Great influence on perfusion and brain oxygenation in early post-traumatic period is caused by micro-rheological blood defection [8], which define its fluidity on capillary level and depend on form, sizes and also erythrocyte membrane condition [9]. It is also known that in the basis of pathological changes, appearing at TBI, there is an activation of free radical oxidation processes, parallel with the decrease in antioxidant protection system activity [3].

The decrease or interruption of continuous generation of antioxidant ability provokes abnormal accumulation of reactive oxygen intermediate implementing the devastating effects at all levels of organisms vital activity [10]. The development of vascular and morphological disturbances are supposed to be one of the most important mechanisms of secondary trauma at that [11, 12]. That is why to stop negative changes, appearing in case of TBI, it is necessary to provide the early correction of hypoxic state and the restoration of cell and subcell membrane homeostasis.

Now a derivative of 3-oxipiridin 2-ethil-6-methil-3-hydroxiperidin succinate is used as a preparation which combines both antihypoxic and antioxidant properties [13]. It is a coordination complex of emoxipin (the derivative of oxipiridin) with amber acid (succinate). This complex is used for correction of neurological and cardiovascular pathologies [14–16]. The research results received in previous studies showed that 2-ethyl-6 -methil-3-hydroxiperidin succinate unlike other succinate antihypoxic drugs enter the cells considerably quicker and then it dissociates in cytosol in two components. Each of them makes an independent positive impact on brain and myocard in ischemia reperfusion thanks to high penetration properties of emoxypine. Emoxypine provokes the inhibition of free-radical processes. Amber acid allows to sustain the processes of high-energy compounds formation [17, 18]. Taking into account this fact it’s possible to suppose that 2-ethil-6-methil-3-hydroxiperidin succinate may play any role in the correction of RBC membrane molecular mechanisms and of cell metabolic status which is the response to the homeostasis system destabilization provoked by TBI. So, it’s impact may improve the perfusion of microvessels. However, the therapeutic potential of this compound to influence the structural functional state of erythron and microcirculation in TBI is not yet studied. As macro- and microrheological disorders of blood–vascular system play a considerable role in extracranial disturbances in TBI the search of drugs which would make a correction of RBCs and of microcirculation is feasible and of grand importance.

The intention of this research was to study if the 2-ethil-6-methil-3-hydroxiperidin succinate injection capable to reduce the risk of claudication in brain cortex as a response to the restoration of functional and metabolic indices of RBCs. The rat model of TBI was used. As it’s difficult to study a mono exposure in medical condition the experiments are made with animals. Such models provide a practical platform for initial evaluations and studies that can inform clinical practice and provide a better understanding of the effects of drugs in the post-traumatic period. In numerous studies it’s proved that the distinction of TBI consequences depend on the sex of animals. It’s shown that women incur a higher risk of adverse outcome in brain commotion than men. So, though men incur a higher risk to receive a brain commotion because they make more often activities associated with risk of brain commotion, women as a rule have more grave consequences of the brain commotion. Nowadays the most studies of people received TBI concern adult men [19, 20]. The preclinical models of trauma concern adult males of animals. It makes a gap concerning the study of female brain and the elaboration of personalized and efficacious methods of TBI treatment.

In this regard, our goal was to study the RBCs functional and metabolic parameters and the brain microcirculatory bloodstream structure under the action of 2-ethil-6-methil-3-hydroxiperidin succinate in order to limit secondary faults modeling female TBI.

## Results

### The analysis of RBCs functional indexes under the action of 2-ethil-6-methil-3-hydroxipiridin succinate.

The animals suffered the experimental TBI had disorders of functional RBC indexes in the post-traumatic acuity (Table 1). From the first day on the background of TBI the rats from the control group demonstrated a considerable decrease in RBC electrophoretic mobility by 14.3% and the increase in RBC aggregation degree by 131.3% relative to the indicators of the intact group of animals. During all the post-traumatic period the RBC electrophoretic mobility increased step by step and the aggregation decreased. But the restoration of the indexes up to the intact group value did not take place. In the groups where 2-ethil-6-methil-3-hydroxipiridin succinate was injected the decrease in RBC electrophoretic mobility was by 24% lower than in that of the control animals from the very first day of the post-traumatic period. On the 3rd day of experiment caused the increase in RBC electrophoretic mobility of EPME by 25.8% and decrease in aggregation degree by 36% in comparison with the control. On the 12th day of the study the RBC electrophoretic mobility and aggregation indices restored up to the normal value.

**Table 1:** The change in functional indexes of RBCs (erythrocytes) in the TBI post-traumatic period of rats in the investigated groups

Value	Intact rats	Group	Period after TBI (day)			
			1	3	7	12
RBC electrophoretic mobility, μm∙cm∙V-1∙s^−1^	1.19 ± 0.03	Experiment	1.02 ± 0.02*^▲^	1.07 ± 0.03*^▲^	1.08 ± 0.03*^▲^	1.11 ± 0.02 *^▲^
		Control	0.78 ± 0.03*	0.85 ± 0.03 *	0.93 ± 0.03 *	1.05 ± 0.02 *
RBC aggregation, %	36.85 ± 1.17	Experiment	87.82 ± 3.79*	49.84 ± 5.81*^▲^	44.22 ± 2.51 *^▲^	38.19 ± 1.18^▲^
		Control	85.81 ± 0.74*	77.89 ± 1.35*	69.09 ± 1.13*	59.68 ± 0.75*
MDA, nmol/ml	0.08 ± 0.01	Experiment	0.20 ± 0.04 *	0.09 ± 0.02 * ^▲^	0.08 ± 0.04 ^▲^	0.10 ± 0.04
		Control	0.23 ± 0.02*	0.18 ± 0.04*	0.19 ± 0.03*	0.14 ± 0.02*
Catalase, units/gHb∙min	28.23 ± 0.66	Experiment	38.63 ± 1.98*^▲^	41.59 ± 1.97*^▲^	29.79 ± 1.34^▲^	34.61 ± 1.58*^▲^
		Control	20.46 ± 0.49*	17.02 ± 0.75*	16.65 ± 0.81*	17.28 ± 1.3*
ATP, μmolPn/mL	1.78 ± 0,17	Experiment	1.27 ± 0.17*^▲^	2.17 ± 0.13	2.72 ± 0.21*	2.85 ± 0.39*
		Control	1,49 ± 0.17	2,28 ± 0.19*	2.56 ± 0.13*	2.37 ± 0.19*
2.3-BPG, μmolPn/mL	2.99 ± 0.25	Experiment	5.16 ± 0,42*	6.68 ± 0,23*^▲^	5.68 ± 0,26*^▲^	4.26 ± 0,28*
		Control	5.22 ± 0.57*	5.12 ± 0,25*	4.01 ± 0,34*	3.79 ± 0,32

Data are means ± SD. “*” statistically important differences regarding the intact group data, p ≤ 0.05, “^▲^” statistically important differences of experiment from control, p ≤ 0.05

These changes were accompanied by the increase in lipid peroxidation (LPO) products quantity and the decrease in catalase activity (by 187.5% and 27.5%, respectively) of the rats from the control group in comparison with the animals from the intact group. Within the following 7 days of post-traumatic period reliably high activity of LPO processes in comparison with the animals from the intact group. This was accompanied by a further decrease in catalase activity by 41.1% on the 7th day after the injury relative to the the intact group animal value. The injection of 2-ethil-6-methil-3-hydroxipiridin succinate provoked the decrease in MDA concentration up to the normal value on the 3rd day of the experiment. Catalase activity was increasing from the 1st day of the post-traumatic period and exceeded the level of the intact group values. The maximal increase in catalase activity (by 144.4%) relative to the control group value was registered on the 3rd day of the post-traumatic period.

The study of ATP and 2.3-BPG concentration in RBCs showed the increase in the indices in all the comparison groups. But it was strongly pronounced where 2-ethil-6-methil-3-hydroxipiridin succinate was injected. On the first day after the trauma, the ATP concentration in the RBCs in the control group was by 28.7% lower and on the 3rd day, it was by 21.9% higher than that of the intact group. The maximal increase of ATP concentration was registered on the 7th day after the trauma. The 2.3-BPG concentration in the RBCs of the control group on the 1st day of the experiment was by 74.5% higher than that of the intact group. On the 1st and 3rd day of the post-traumatic period, after the 2-ethil-6-methil-3-hydroxipiridin succinate injection the ATP concentration value in RBCs was lower than that of the intact animal group. On the 7th and 12th day the ATP concentration was higher by 34.1% and 26.8% respectively than that of the intact group. From the 3rd day of the experiment till its end the 2.3-BPG concentration was higher. So, On the 3rd, 7th, and 12th day of the experiment it was higher by 123.4%, 90% and 49.2% respectively than that of the intact animal group.

### Microcirculatory mainstream morphofunctional changes at TBI and under the action 2-ethil-6-methil-3-hydroxipiridin succinate

**Fig. 1:**
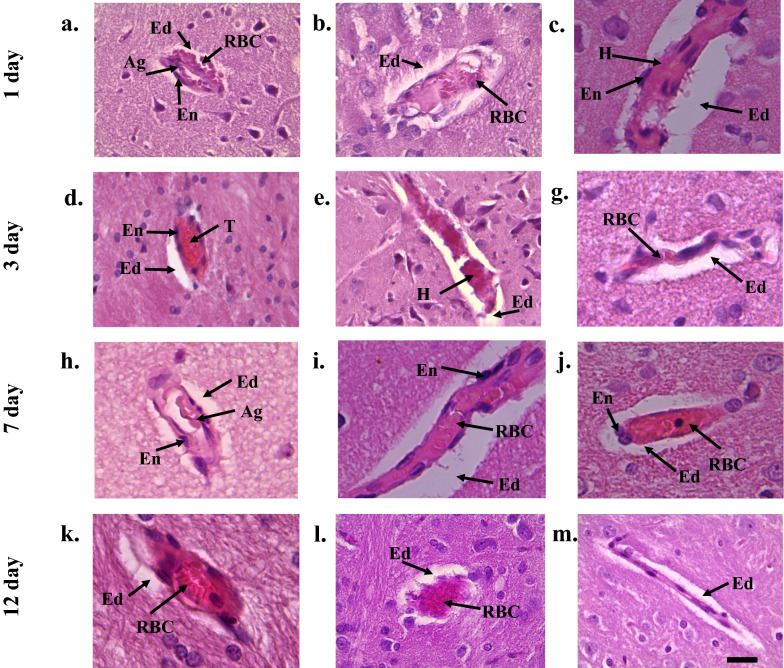
The structure of brain microcirculatory mainstream of the rats from control group after traumatic brain injury. (**a** Arteriole. One day after the TBI vascular fullness, single perivascular hemorrhages, aggregates of erythrocytes were observed and perivascular edema was highly expressed, **b**—Venule. One day after the TBI, diapedesis, stored erythrocytes were observed and as well as highly expressed perivascular edema, **c**—Capillary. One day after the TBI in brain spawn capillaries the erythrocytes were absent. In some capillaries they were like sludges. Also everywhere there was highly expressed perivascular edema, **d**—Arteriole. 3 days after the TBI in arterioles clearance there were pink hyaline thrombuses, highly expressed perivascular edema was formed, **e**—Venule. 3 days after the TBI sludge erythrocytes and hyaline like masses, highly expressed endothelium edema and prevascular edema were noted in venules clearances, **f**—Capillary. 3 days after the TBI sludge erythrocytes, endothelium edema, highly expressed perivascular edema were noticed in the clearance, **g**—Arteriole. 7 days after the TBI in arterioles there were the parietal erythrocytes aggregations. Moderate endothelium edema was observed and perivascular edema was highly expressed, **h**—Venule. 7 days after the TBI in venules there was moderate endothelium edema, in the clearance between them there were protein masses, slagged of erythrocytes and blood clots of erythrocytes, moderately expressed perivascular edema was defined everywhere, **i**—Capillary. 7 days after the TBI in capillaries plethora, highly expressed endothelium and perivascular edema were noted, **j**—Arteriole. 12 days after the TBI in arteriole lumen there were erythrocytes and moderate perivascular edema, **k**—Venule. 12 days after the TBI free laying erythrocytes, endothelium edema was not expressed, moderately expressed perivascular edema was defined were defined, **l**—Capillary. 12 days after the TBI in the capillary lumen there were free laying erythrocytes, moderate endothelium edema and moderate perivascular edema). Scale bar is 100 μm, original magnification × 200 for arteriole and venule. Scale bar is 50 μm, original magnification × 400 for capillary. Abbreviations: Ag—aggregates from red blood cells, Ed edema, En endothelium, H—hyaline blood clots, RBC—red blood cells, T thrombuses

The of histological investigations of animals in the post-traumatic period showed that one day after the TBI there were small-focal hemorrhages, pronounced perivascular edema, vasodilation and fullness of blood vessels in the area of the primary trauma (Fig. 1a–c). In particular, vascular fullness with manifestations of stagnation was observed in the vessels of the microcirculatory bed and veins (Fig. 1b, c). 70% of venules and 30% of the arterioles were occluded by microthrombs. Outside the focus of primary damage, there was an uneven blood filling of blood vessels, a complete decline in the lumen of some and overflow of blood of others, edema of the vascular wall and endothelial cells. The above changes were visualized at the level of arterioles, veins, and capillaries.

In some cases, there was a rupture of the integrity of the vascular wall and the exit of erythrocytes outside the vascular bed diapedesis (Fig. 1a, b). In the lumen of the arterioles, there were blood clots and aggregates from erythrocytes, and strongly expressed perivascular edema (Fig. 1a). In the lumen of the venules, diapedesis, slags and parietal aggregates from red blood cells were observed, and strongly expressed perivascular edema (Fig. 1b). The area of perivascular edema of venules and arterioles relative to the norm was higher by 5.6 and 3.8 times, respectively, with the predominance of changes in the zone of primary damage (Table 2).

**Table 2:** Characteristics of morphometric indicators of the microcirculatory bed of the brain in the investigated groups of rats (M + m)

Value	Intact rats	Group	Period after TBI (day)			
			1	3	7	12
Diameter of the capillary, µm^2^	5.5 5 ± 0.06	Control	3.25 ± 0.05*	3.95 ± 0.06*	5.21 ± 0.05*	5.94 ± 0.03*
		Experiment	3.53 ± 0.04*	3.49 ± 0.05*	4.75 ± 0.03*^▲^	5.19 ± 0.04*^▲^
Area of the capillary, µm^2^	41.9 ± 5.31	Control	114.86 ± 12.32*	154.64 ± 10.41*	129.69 ± 9.87*	109.06 ± 34.51*
		Experiment	120.62 ± 9.85*	124.28 ± 8.63*^▲^	98,.6 ± 9.45*^▲^	45.02 ± 6.21*^▲^
Area of pericapillary edema, µm^2^	124.2 ± 12.83	Control	375.79 ± 27.51*	533.86 ± 34.71*	277.04 ± 28.08*	194.64 ± 24.18*
		Experiment	345 ± 26.82*	262.82 ± 14.54 *^▲^	144.29 ± 11.05^▲^	117.75 ± 12.47^▲^
Area of the capillary bed, µm^2^	628 ± 32.24	Control	456 ± 26.01*	854 ± 41.64*	748 ± 34*	560 ± 29*
		Experiment	904.65 ± 38.32*^▲^	1 118.2 ± 35.26*^▲^	884 ± 31*	630 ± 33^▲^
Area of the venules, µm^2^	185 ± 14.21	Control	335.62 ± 20.14*	356.05 ± 19.32*	293.67 ± 18.41*	254.06 ± 17.64*
		Experiment	328.91 ± 21.01*	282 ± 17.64 *^▲^	22,658 ± 15.32*^▲^	205.17 ± 15.08^▲^
Area of perivascular edema of venules, µm^2^	124.2 ± 12.83	Control	801.75 ± 21.51*	906.24 ± 25.68*	574.28 ± 22.56*	391.26 ± 19.42*
		Experiment	774.08 ± 24.13*^▲^	504.78 ± 21.25*^▲^	308.2 ± 17.34 *^▲^	178.35 ± 14.72 *^▲^
Area of the arterioles, µm^2^	320 ± 15.2	Control	458 ± 25.45*	471 ± 26.81*	421 ± 18.21*	408 ± 16.14*
		Experiment	452 ± 24.62*	403 ± 21.51*^▲^	373 ± 18.93 *^▲^	306 ± 14.72 *^▲^
Area of perivascular edema of arterioles, µm^2^	243.2 ± 14.72	Control	925.16 ± 25.16*	1059.75 ± 21.5*	719.91 ± 22.14*	510 ± 20.05 *
		Experiment	899.48 ± 19.63*	834.19 ± 17.64*^▲^	514.78 ± 15.64*^▲^	266.22 ± 16.35^▲^
Swelling of the neuron, µm^2^	140.43 ± 8.74	Control	251 ± 12.36*	264 ± 11.04*	220 ± 12,31*	206.66 ± 8,84*
		Experiment	208.85 ± 9.75*^▲^	204.7 ± 10.62*^▲^	168 ± 7.84*^▲^	136.25 ± 5.64*^▲^

Data are means ± SD. “*” statistically important differences regarding the intact group data, p < 0,05, “^▲^” statistically important differences of experiment from control, p < 0,05

In the dormant capillaries there were no RBCs, in some fields of vision in their lumen, red blood cells were located in the form of aggregates and slags (Fig. 1c). In all capillaries, an increase in the area of pericapillary edema by 2.7 times relative to the norm was observed. In comparison with intact rats, there was a decrease in capillary density by 15–20%, a decrease in their diameter by 40%, and as a result, a decrease in the area of the capillary bed in halve.

The maximum changes in the morphological state of the vascular bed of the brain were observed on the 3rd day after the injury. The arterioles showed a visible edema of the endothelium, and red and hyaline blood clots were found in their lumen (Fig. 1d). The venules detected pronounced endothelial edema and stored RBCs and hyaline-like masses were detected in the lumen (Fig. 1e). At the same time, the area of perivascular edema of arterioles and venules was 14% and 13% larger, respectively, compared to the 1st day after the injury. The microcirculatory bed retained narrowed capillaries with strongly pronounced pericapillary edema (Fig. 1f), but in comparison with the 1st day after injury, there was an increase in the area of the capillary bed.

7 days after the injury, there was a significant tendency in the control group of animals to decrease the area of the capillary bed against the background of an increase in the average diameter of capillaries relative to the 3rd day value. At the same time, the capillaries remained full of blood, strongly expressed endothelial and perivascular edema (Fig. 1i) took place. Parietal aggregates of RBCs were found in arterioles, moderate edema of the endothelium and strongly expressed perivascular edema were determined (Fig. 1g). In the lumen of some venules, free-lying red blood cells were detected, but in most venules, protein masses, slagged red blood cells and blood clots, moderate endothelial edema and perivascular edema were detected (Fig. 1h).

On the 12th day of the study, the control group of animals showed a restoration of blood circulation in the cerebral cortex. Free-lying red blood cells were detected in the lumen of arterioles (Fig. 1j) and venules (Fig. 1k), endothelial edema was not expressed, but single blood clots and moderate perivascular edema persisted everywhere. The area of perivascular edema of arterioles decreased in halve, and venules by 2.24 times relative to the values for 1 day. When examining the capillary bed, it was found that free-lying red blood cells were observed in the lumen of the capillaries, moderate endothelial edema and moderate perivascular edema were preserved (Fig. 1l). The average diameter of capillaries was higher by 8% relative to the indicators of the intact group, the density of the capillary bed was lower by 28% relative to the indicators on the 7th day, but was 10% lower than normal (Fig. 1m).

**Fig. 2:**
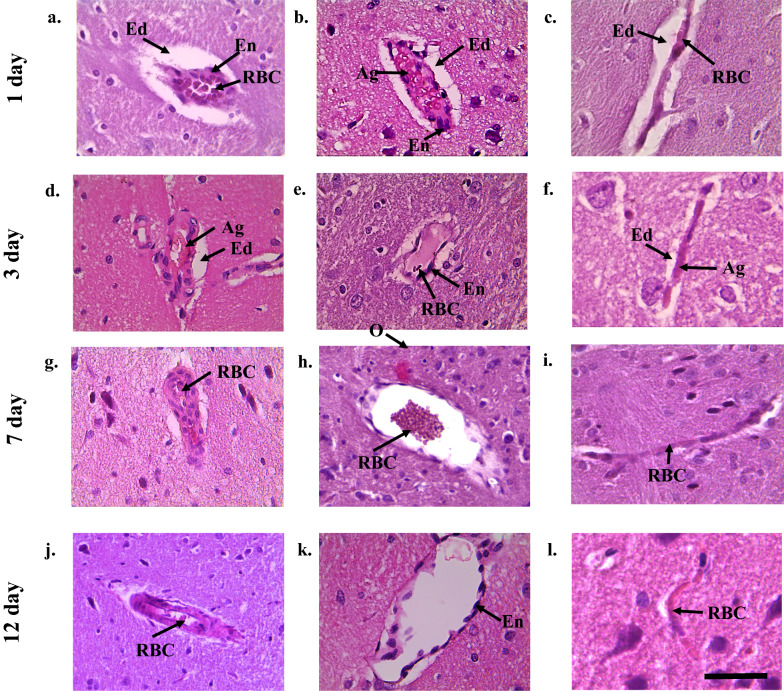
The structure of brain microcirculatory mainstream of the rats after the traumatic brain injury under the action of 2-ethil-6-methil-3-hydroxiperidin succinate. **(a** Arteriole. Within 1 days after TBI in arteriole clearance there were singular erythrocytes, moderate endothelium edema, highly expressed perivascular edema, **b**—Venule. 1 day after the TBI in venule lumen there were erythrocyte aggregations, moderate endothelium edema, highly expressed perivascular edema, **c**—Capillary. 1 day after TBI in capillary lumen there were sludge from erythrocytes, moderate endothelium edema, highly expressed perivascular edema, **d**—Arteriole. 3 days after the TBI there were the parietal erythrocytes aggregations in arterioles. Moderate endothelium edema and perivascular edema were defined, **e**—Venule. 3 days after the TBI there were moderate endothelium edema, in the lumen between them they were defined, **f**—Capillary. 3 days after the TBI in capillaries erythrocytes sludges, moderate perivascular edema were defined, **g**—Arteriole. 7 days after the TBI the arteriole lumen partially collapsed, with singular erythrocytes in its lumen. Poorly expressed perivascular edema was observed, **h**—Venule. 7 days after the TBI here were distinguished free laying count blood elements in the lumen of the most of venules. Everywhere poorly expressed perivascular edema was observed, **i**—Capillary. 7 days after the TBI there were free laying erythrocytes in the capillary lumen, perivascular edema was not expressed, **j**—Arteriole. 12 days after the TBI there were singular count blood elements in the most of arteriole lumens; endothelium was without the features of edema. Perivascular edema was poorly expressed, **k**—Venule. 12 days after the TBI there were no count blood elements in the lumens of the most of venules or it was possible to meet singular free laying erythrocytes. Endothelium was without or with poorly expressed edema. Perivascular edema was poorly expressed, **l**—Capillary. 12 days after the there were free laying erythrocytes in capillary lumens. Around the most of them perivascular edema was absent). Scale bar is 100 μm, original magnification × 200 for arteriole and venule. Scale bar is 50 μm, original magnification × 400 for capillary. Abbreviations: Ag—aggregates from red blood cells, Ed edema, En endothelium, H—hyaline blood clots, RBC—red blood cells, T thrombuses

**Fig. 3:**
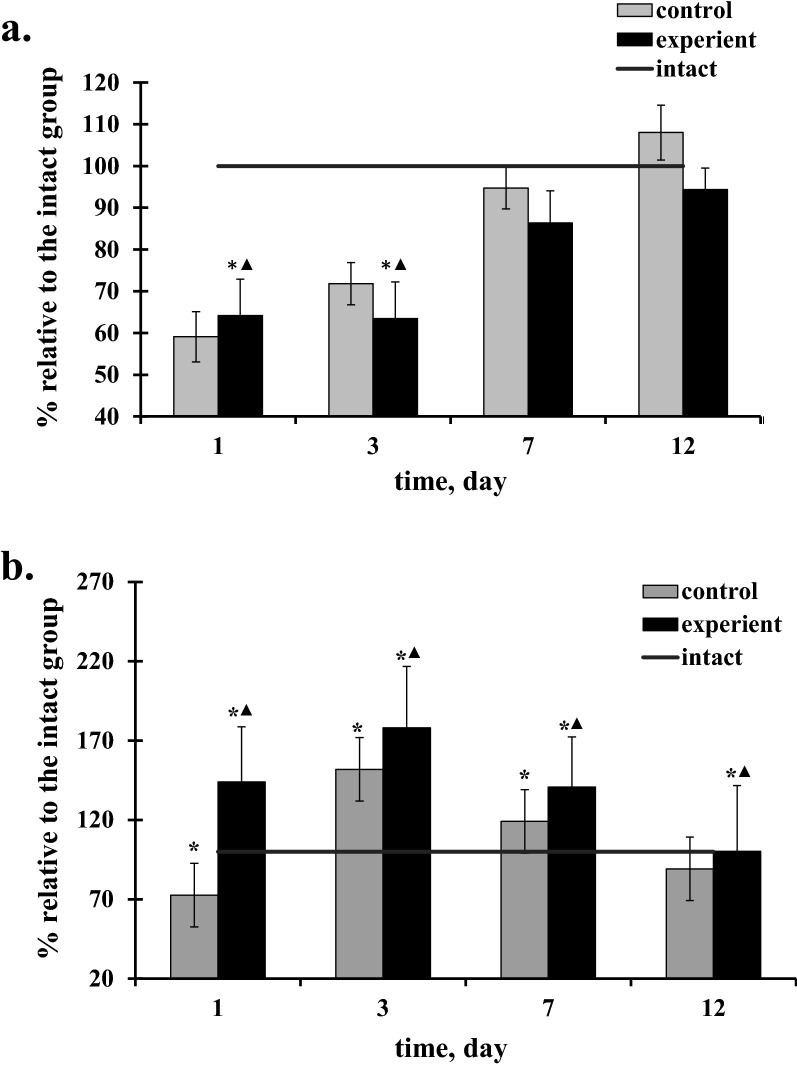
Effect of 2-ethil-6-methil-3-hydroxiperidin succinate treatment on the average capillary diameter (a) and area of the capillary bed (b). Data are means ± SD. “*” statistically important differences regarding the intact group data, p < 0,05, “▲ “ statistically important differences of experiment from control, p < 0,05

1 day after the TBI in animals of the experimental group were observed vacuolation of the intercellular space, vessels of the microcirculatory bed with less pronounced aggregation of red blood cells, but strongly expressed edema of the endothelium and perivascular edema, as well as in the control group (Fig. 2a–c). Free-lying red blood cells were detected in the lumen of most arterioles (Fig. 2a), wall aggregates of red blood cells and microthrombs were observed in 10% of arterioles. In the lumen of the venules, aggregates of red blood cells, parietal located hyaline-like masses were recognized (Fig. 2b). The area of perivascular edema of arterioles and venules was 3.7 and 5.4 times higher than those of the intact group, but by 2% and 5% lower than that of the control group (Table 2). In comparison with the animals of the intact group, there was a decrease in the average capillary diameter (Table 2, Fig. 3a). It should be noted that despite of the narrowing of the capillary lumen after the injury, most capillaries are in a functionally active state, which is confirmed by morphometric analysis, indicating that the area of the capillary bed was statistically significantly higher after the treatment with the action of 2-ethyl-6-methyl-3-hydroxypyridine succinate compared to similar values in the control group of rats (Table 2, Fig. 3b).

On the 7th day of the post-traumatic period, the experimental group showed a visible reparative changes with a tendency to normalize the structure of the microcirculatory bed. Free-lying red blood cells were detected in the lumen of arterioles and venules, edematous endothelium was observed of vessels, and weakly expressed perivascular edema was detected everywhere (Fig. 2g, h). The diameter of the capillaries and the area of the capillary bed were restored to normal values in spite of the general tendency on against the background of a decrease in pericapillary edema (Fig. 2i; Table 2). Reduction of pericapillary edema is associated with restoration of the structural integrity of the endothelial lining. By the 12th day of the study, the lumen of the arterioles had no formed blood elements, vascular endothelium without signs of edema, and perivascular edema was poorly expressed (Fig. 2j). In the lumen of most venules, there were no shaped blood elements or there were single free-lying red blood cells, the endothelium was slightly edematous, and perivascular edema was weakly expressed (Fig. 2k). In the capillaries, the diameter did not differ significantly from the norm, free-lying red blood cells were detected in the lumen, and there was no pericapillary edema (Fig. 2l).

### Morphofunctional changes of brain tissue with traumatic brain injury and action of 2-ethil-6-methil-3-hydroxipiridin succinate.

**Fig. 4:**
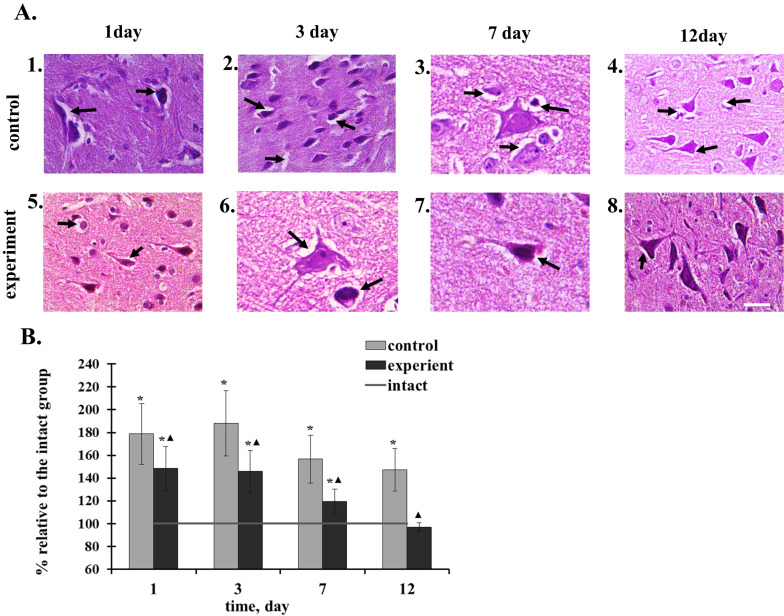
Effects of 2-ethil-6-methil-3-hydroxypiridin succinate on Neurons and glial cells of rat brain tissue after the traumatic brain injury. (A) Neurons and glial cells of control rats and rat under the action of 2-ethil-6-methil-3-hydroxypiridin succinate. Scale bar is 50 μm, magnification × 400. (B) Quantification of pericellular edema around the neurons and glia cells. (**1** on the first day of post-traumatic period, there was a strongly pronounced pericellular edema around neurons and glia cells. The intercellular substance had an almost frothy appearance; **2** on the 3rd day of post-traumatic period there was a strongly expressed pericellular edema around neurons and glia cells. Moderate amount of vacuoles was in the intercellular substance; **3** on the 7th day of the post-traumatic period edema around the neurons and glia cells was moderate. Small number of vacuoles was in the intercellular substance; **4** on the 12th day of the post-traumatic period there was was a moderate pericellular edema around neurons and glia cells. Single vacuoles were in the intercellular substance; **5** on the 1st day of the post-traumatic period there was a severe pericellular edema around neurons and glia cells. Moderate amount of vacuoles was in the intercellular substance; **6** on the 3st day of the post-traumatic period there was a moderate pericellular edema around neurons and glia cells. Moderate amount of vacuoles was in the intercellular substance; **7** on the 7th day of the post-traumatic period there was a weakly expressed pericellular edema around neurons and glia cells. Single vacuoles were in the intercellular substance; **8**—on the 12 day of the post-traumatic period the pericellular edema around the neurons and glia cells was absent. The intercellular substance was not vacuolated). Examples of pericellular edema around the neurons and glia cells are indicated by arrows

Morphofunctional changes after the TBI were characterized by degenerative-dystrophic changes in the control group of rats (Fig. 4). The maximum changes were observed on the 1st and 3rd days. One day after the TBI, the total number of neurons was lower than that in the intact group of animals. Expressed dystrophic changes were observed in neurons, part of them were swollen, very few shrunken, with poorly distinguished nucleus and nucleolus, homogeneous cytoplasm and with twisted deformed scions. There were also detected single neurons with indistinct contours, with progressive karyoplasmocytolysis and almost lost nucleus (Fig. 4A). The area of neuron edema was 80% larger compared to the norm (Table 2, Fig. 4B). There was also visible and pronounced pericellular edema around the glia elements.

On the 3rd day of the post-traumatic period pronounced dystrophic changes were recognized in neurocytes: neurons with karyolysis, in the state of neurocytolysis, with vacuolated cytoplasm, deformed cell membrane and strongly expressed pericellular edema. General neuron density was low, but the number of shrivelled hyperchromic neurons and cells-shadows increased. Glial cells are light of rounded form with intensively colored nucleolus, the pericellular edema persisted around them (Fig. 4A). It should be noted that the bulk of the altered neurons was located in areas which were far from the capillaries. In the brain tissue in the area of primary damage vacuolation and expansion of perivascular and perineuronal spaces was observed, which reflected the phenomena of diffuse brain edema.

On the 7th day in the control group of animals a part of neurons were preserved in a swallen state, with karioreksis in the form of “melting” neurons and cells-shadows. The area of pericellular edema decreased by 12% compared to the 1st day value. Glial cells preserved the correct body form, sometimes have the jagged edges, their nucleus was colored intensively, severily expressed pericellular edema was noted around them (Fig. 4A). On the 12th day of the investigation the restoration of brain tissue was observed, the decrease in pericellular spaces, edema spaces around neurons by 30%, the number of hyperchromic shriveled neurons and shadow cells relative to 3rd day of the study took place (Fig. 4A).

On the 1st day after TBI the animals from the experimental group demonstrated hydropic changes in brain cells. In the clarified cytoplasm there was spongy foam, separate vacuoles, the nuclei were under the influence of erosion, of a changed form; clarified or pictonic, some of them lost the nucleolus. The general neuron density was lower relative to the norm, but compared to the control, the loss of neurons was less pronounced. Highly expressed pericellular edema was formed around glial cells and neurons (Fig. 4A).

On the 3rd day of the post-traumatic period the structural changes of brain tissues were less expressed in the experimental group in comparison with the control group: poorly expressed perinuclear edema and moderate pericellular edema took place. Neurons were with well expressed nucleus and nucleolus, there were long protrusions, light glial cells of rounded form with intensively colored nucleolus (Fig. 4A). On the 7th day after the trauma, the processes of regenerative reparation in different intensity were observed.

This is evidenced by the preservation of most neurons with a well-defined nucleus, nucleolus and long processes. The area of pericellular edema decreased by 20% relative to the 1st day value and by 23% relative to the control group value (Fig. 4B). In addition, there were signs of compensatory-restorative reactions of preserved neurons and glia cells, which were structurally manifested at the neuron level by restoring the tinctorial properties of the cytoplasm, hypertrophy of the soma and nucleus with a decrease in the nuclear-cytoplasmic ratio and quality characteristics of supporting cells (astrocytes). In the absence of widespread foci of destruction and hemorrhagic infection, hyperchromic shrunken neurons and shadow cells were found only in places. Around the neurons and glial cells moderate and poorly expressed pericellular edema was noted (Fig. 4A).

On the 12th day of the post-traumatic injury neurons and glial cells the reconstruction of neurons and glial cells in animals was observed. Here and there poorly expressed perinuclear edema was preserved (Fig. 4A).

### Animal motion activity analysis

Tonic and clonic seizures had been observing just after the TBI for 2–4 s. They lost sensitivity and they had been in lateral position for 10-20 s. The study of motor response by method of moving on bar showed the worsening of motor function up to the 1st of the experiment (Table 3).

**Table 3:** Motion activity analysis in the TBI post-traumatic period of rats in the investigated groups

Value	Intact rats	Group	Period after TBI (day)			
			1	3	7	12
Moving on bar, numbers	2.5 ± 0.1	Control	6.8 ± 0.9*	5.7 ± 0.9*	4.9 ± 0.5*	4.5 ± 0.7*
		Experiment	4.2 ± 0.8*^▲^	4.0 ± 0.4*	2.9 ± 0.3^▲^	2.6 ± 0.4^▲^
Paw sliding frequency, numbers	1.0 ± 0.1	Control	3.6 ± 0.7*	2.8 ± 0.6*	2.0 ± 0.5	2.2 ± 0.5*
		Experiment	1.6 ± 0.7*	1.3 ± 0.4*	1.1 ± 0.2*	0.8 ± 0.2^▲^
Time of moving on bar, numbers	1.5 ± 0.1	Control	1.6 ± 0.7*	1.3 ± 0.4*	1.1 ± 0.2*	0.8 ± 0.2^▲^
		Experiment	1.8 ± 0.2^▲^	1.6 ± 0.2^▲^	1.4 ± 0.2^▲^	1.4 ± 0.2^▲^

Data are means ± SD. “*” statistically important differences regarding the intact group data, p < 0.05, “^▲^” statistically important differences of experiment from control, p < 0.05

2-ethyl-6-methyl-3-hydroxypyridine succinate injection provoked the normalization of standing balance and walking ability. It manifested itself in the decrease in paw sliding frequency as well as in time spent for moving on bar. The paw sliding frequency and time of moving on bar decreased by 55.5% and 61.7% correspondingly by the end of the first day in the group treated with 2-ethyl-6-methyl-3-hydroxypyridine succinate. The positive dynamics in the motion activity indices was evident to the end of the first day after the 2-ethyl-6-methyl-3-hydroxypyridine succinate injection. A considerable improvement of motion reactions were registered by the 3^d^ day after the injection. The level of these indices achieved the intact animal value to the 7th day after the 2-ethyl-6-methyl-3-hydroxypyridine succinate injection. In the control group recovery was observed only in 12 days.

## Discussion

The research results demonstrates that the application of 2-ethil-6-methil-3-hydroxipiridin succinate after TBI already on the 3rd day increased RBC electrophoretic mobility, decreased aggregation level and the intensity of LPO processes in RBCs. The decrease in RBC aggregation is caused by the increase in their electronegativity, which is restored under the influence of 2-ethil-6-methil-3-hydroxipiridin succinate. May be it’s possible that one of the mechanism of the charge conservation which took place in the experiment is the increase in the stability of membrane structure caused by the use of drug, it has been shown for several times that the stability of the membrane is largely recognized by LPO processes [21, 22]. It is discovered that emoxipin which is the constituent of a given preparation, inhibit free radical oxygenation, cooperates actively with peroxide lipid radicals, hydroxyl peptide radicals, stabilizes cells membranes. Besides, the effects of 2-ethil-6-methil-3-hydroxipiridin succinate may be mediated, as we believe, by the presence of succinate in the preparation. In particular, it has been shown that the use of succinate reduces the formation of malondialdehyde [23]. In addition, it has been shown that when the intracellular oxygen concentration decreases and/or the rise of succinate concentration HIF-1a factor activity increases [24]. HIF-1a is an oxygen-sensitive protein complex that triggers the expression of a number of peptides, including erythropoietin (EPO), glucose Transporter proteins (GLUT 1, 3), and glycolysis enzymes [25]. We suppose that in this case, there may be an increase in erythropoiesis and the formation of erythroblasts with high energetic potential on the early stages of erythropoiesis with the following way out of mature RBCs resistant to hypoxia to the vascular bed. It is also demonstrated that an increase in extracellular succinate concentrations through the interaction of GPR91-receptors associated with G-proteins leads to the activation of intracellular metabolism [26].

The results given in the Table 1 prove the given statement. The content of 2.3-BPG and ATP under the action of 2-ethil-6-methyl-3-hydrospiridin succinate from the 3rd and the 7th day of the experiment was higher than that of the control group respectively (p ≤ 0,05). The content of ATP and 2.3-BPG defines the deformation of RBCs which influenced the RBCs aggregation [27], which blocks the blood flow and provokes the disorder of transcapillary exchange [28, 29] 2-ethil-6-methil-3-hydrospiridin succinate caused the increase of metabolic and the restoration of oxidizing processes, which defined the decrease in RBC aggregation.

Besides, RBCs transporting oxygen to brain tissues, depending on their functional activity, influence greatly the degree of tissue hypoxia expression [30]. We note that 2.3-BPG acts as an important allosteric regulator of binding oxygen with hemoglobin [31]. The increase in 2.3-BPG production of RBCs, obtained in our experiments, facilitate the release of oxygen in tissues which promotes the content of pO_2_ in blood and tissues on the adequate level.

It should be noted that with the action of 2-ethyl-6-methyl-3-hydroxypyridine succinate, there is an increase in the area of the capillary bed, probably due to the inclusion of plasma capillaries, which were involved in response to the increased oxygen demand of the brain in the post-traumatic period. In addition, an increase in succinate concentration activates HIF-1a, which causes an increase in endothelial growth factor (VEGF) [25]. VEGF acts selectively on vascular endothelium, ensuring its stability, promoting proliferation, migration and formation of endothelial cell tubules, that affects angiogenesis. Reduced VEGF levels cause endothelial apoptosis, leading to vascular lumen obstruction [32]. Maintaining the endothelium in a stable state also contributes to NO. VEGF interacts with endothelial NO-synthase (eNOS) in the caveoles of endothelial cells, regulating its activity and thereby contributing to the production of NO. NO is a powerful vasodilator [33]. It is likely that the marked pathways of succinate influence through VEGF may also occur with the action of 2-ethyl-6-methyl-3-hydroxypyridine succinate in the post-traumatic period of TBI.

The increase in oxygen-transport blood function and of the capillary bed in post-traumatic period of traumatic brain industry revealed in the group of animals in which 2-ethyl-6-methyl-3-hydroxypyridine succinate was injected is an important result of the investigation as it is demonstrated that a distinctive feature of secondary disorders of TBI is microvascular disorders which happens immediately after the primary mechanical trauma [10, 34, 35].

At the same time, rapid and timely restoration of blood flow in the penumbra zone ensures cell survival and restoration of neurological deficits. In its turn, in spite of the fact that the structural and functional integrity of the endothelial lining restored, there was a decrease in edema and restoration of neurons and glia in the post-traumatic period. Probably, the positive effect of succinate on neurons after TBI can be realized through GPR91 receptors, which are found in the kidneys, liver, blood cells, adipose tissue, retina, and nervous tissue [36, 37] The expression of GPR91 mRNA and the protein itself is recognized in neurons and astrocytes of the cortex [38]. Activation of GPR91 leads to an increase in intracellular Ca^2+^ [26], which is a secondary messenger and largely determines the metabolic activity of cells.

The investigations of microcirculatory mainstream and brain tissues proves the validity of a given statement. The comparative analysis of the results of the functional state of RBCs, morphological investigation of microcirculatory mainstream and brain tissues of rats suffering from TBI, allows to come to a conclusion that intraperitoneal continuous injection of 2-ethil-6-methil-3-hydroxipiridin succinate on the early stages prevents or / and decreases micro vessels, neurons and glia disorder in a post-traumatic period. It is expressed in an earlier hemorrhage disappearance, the decrease in pericellular, intracellular and pericircular edema development, the preservation of morphology of microcirculatory mainstream and nervous tissue in comparison with control animals.

Taking into account, the widespread prevalence of TBI and the fact that today there is no adequate treatment [20], the results of our experimental work show the effectiveness of the use of 2-ethyl-6-methyl-3-hydroxypyridine succinate in the correction of traumatic brain injuries. Confirmation of the restoration of brain homeostasis was obtained in the study of the functional state of animals: the study of motor reactions of rats in the test "method of movement on a bar" revealed a significant reduction in the time of manifestation of motor dysfunction when using 2-ethyl-6-methyl-3-hydroxypyridine succinate compared to the control compared to the control. From the point of view of further directions of research, it would be advisable to investigate whether the revealed effects of 2-ethyl-6-methyl-3-hydroxypyridine succinate are specific for females with TBI or are they stereotyped. Because on the one hand, the composition suggests the non-specificity of the action of 2-ethyl-6-methyl-3-hydroxypyridine succinate on erythrocytes and the morphology of the microvasculature of animals, on the other hand, a marked more aggressive neuroinflammatory profile in males compared to females during acute and subacute phases after injury suggests a different intensity of the effects [19].

### Findings

1. The use of 2-ethil-6-methil-3-hydroxipiridin succinate determined the decrease in oxidative stress in animals and RBC aggregation, the increase in electro negativity and ATP and 2.3-BPG concentration from the 3rd till the 7th day of TBI.
2. The use of 2-ethil-6-methil-3hydroxipiridin succinate demonstrated the restoration of the state of microcirculatory mainstream beginning on the 7th day of post-traumatic injury whereas in control the restoration processes were registered on the 12th day.
3. The animals protected by 2-ethil-6-methil-3-hydroxipiridin succinate demonstrated the restoration of the structures of brain nervous tissue after TBI quicker than those of the control group.

## Conclusions

The improvement of RBC functional metabolic indices and of histologic indices of microcirculatory bloodstream and of brain tissues in the post traumatic period of TBI under the influence of 2-ethyl-6-methyl-3-hydroxypyridine succinate injection is an important research result.

Restoring blood flow reduces damage to brain tissue and has a positive effect on angio- and cytoarchitectonics of the cerebral cortex in the post-traumatic period. Revealing the mechanisms of action of 2-ethyl-6-methyl-3-hydroxypyridine succinate using visualization methods allows to substantiate the direction of action of 2-ethyl-6-methyl-3-hydroxypyridine succinate. Thus, the results of the study showed that 2-ethyl-6-methyl-3-hydroxypyridine succinate, having energy-synthesizing, antiradical and oxygen-supplying effects, in the post-traumatic period caused by TBI, has a corrective effect not only on microcirculation, but also on the vascular system, which largely prevents secondary brain damage.

## Materials and methods

### Animals

White females rats weighting 180 ± 20 g the age 14 weeks were obtained from the SPF the vivarium Institute of Biology and Biomedicine of Lobachevsky State University. Rats were kept in a vivarium equipped in accordance with the requirements of the «Sanitary rules for the design, equipment and maintenance of experimental and biological clinics (vivariums)" (SR 2.2.1.3218–14) (Resolution of the Chief State Sanitary Doctor of the Russian Federation of 29.08.2014 № 51). The animals were kept in the same plastic cages with drinkers, and were provided with complete extruded mixed fodder and a sufficient amount of water. Rat were housed in groups of 4–5, with standard rodent chews and water freely accessible during the entire experimental period. Animals were maintained in an artificial 12-h day/night lighting cycle (lights on at 07:00) at a constant temperature of 21 ± 2 °C (50 ± 5% humidity).

The research was conducted in accordance with the rules of work and the use of experimental animals (Annex to the Order of the Ministry of Health of the USSR of 12.08.77 № 775), the European Convention for the Protection of Vertebrate Animals Used for Experimental or Other Scientific Purposes of 18 March 1986 and the requirements of the Order of the Ministry of Health of the Russian Federation of April 1, 2016 № 199n "On Approval of the Rules of Good Laboratory Practice". The research protocol was approved by the Local Ethics Committee for conducting scientific research involving animals as research objects of the Lobachevsky State University on July 4, 2014.

### Animal model and drug administration

One of the most widely used models to replicate focal cerebral contusion as well as diffuse brain injury characterized by axonal damage is weight drop models in rodents. This model induces a TBI by a standardized weight-drop device inducing a focal blunt injury over an intact skull without pre-injury manipulations [39]. Such a mechanical impact on the closed skull, rather than on the exposed dura, certainly mimics, e.g., a fall, a motor vehicle ejection or a motorcycle crash, much more closely than initial trephination of the skull with prolonged exposure of the meninges before impact, cryogenic brain injury, direct suction injury, or a fluid wave striking the exposed brain [40]. In order to simulate the TBI, rats, no anesthetized were situated under a device consisting of a metal tube (inner diameter 20 mm) placed vertically over the animal’s head. Rats were fixed on a tablet, but the head was not fixed. The fall of the load (100 gr) was guided by a cylindrical tube 100 cm long, which was rigidly fixed on a tripod by two holders and centered over the head of the rat [41]. Immediately after the injury, rats were transferred to a special plastic cage, and they were monitored until normal behavioral patterns were restored. The application of such mechanical energy provided simulation of concussion, focal injuries, including brain injury, which is accompanied by the formation of epidural and subdural hematomas. The mortality rate of falling weight was 0–10% and a righting reflex time was 2—4 min. This indicates the development of minor trauma [42]. After the injury, the animals experienced asphyxia, convulsions, bleeding, etc. 30 to 40 min after the injury, the animals returned to normal life and nutrition. The animals that died (n = 2 in total) during the experiments were discarded from this study.

The animals (n = 72) were randomly divided into: the rats that had been being introduced to 2-ethil-6-methil- 3hydroxipiridin succinate (commercial name—mexicor, which is the solution for intra- vein and intra-muscular application, OOO “EcoFarmInvest”, Moscow) at 8 mg/kg intro-abdominally for 10 days every day (experimental group, n = 32) and rat introduced the physiological solution in the same volume (control group, n = 32) after the TBI. The first application of the preparation was 1 h after causing the TBI. The values of the studied parameters in the intact animal group were accepted as values of the physiological norm (n = 8 rats).

### Blood collection and RBCs preparation

Blood samples were collected at each time point from 8 randomly selected rats 1, 3, 7, and 12 days after the TBI. Approximately 2.0 ml whole blood was drawn from the sublingual vein. The first blood sampling was carried out 1 h after the injury. Blood samples were collected in conical tubes containing 1.0 ml of 3.2% buffered sodium citrate solution. For example, in the tube labeled “C” there was blood from one rat suffered the TBI and received the physiological solution, “M”—blood from one rat suffered the TBI and received 2-ethil-6-methil- 3hydroxipiridin succinate; and “I”—another one rate which did not receive any exposure. Cells and plasma had been being separated by centrifugation at 1000 × rpm for 10 min with 0.9% sodium chloride solution.

### RBCs functional indexes analysis

RBC electrophoretic mobility (EPME) was defined with the use of micro-electrophoresis method (n = 8/group/time point) [43]. The time of the distance 100 mkm in Tris–HCL-buffer with pH 7.4 with currant force 8 mA covering was registered. The value of RBC electrophoretic mobility was calculated with the formulae U = S/TH, where S—the distance of cells movement, T—the time of cells movement on the distance S, H—gradient potential. The value of the gradient potential was defined by the formulae H = I/gχ, where I—amperage, g—camera cross section, χ—electrical conductivity of the medium.

RBC aggregation was studied by optic microscopy method by calculating solar erythrocytes and their aggregation (n = 8/group/time point) [44]. As the aggregation stimulator the solution of blue dextran-T-2000 (GE Healthcare firm, 20 mg/ml) was used in Tris HCL- buffer (pH-7.4) was used. Drench RBC diluted with the dextran solution (in proportion 1:10 volume units) and counted the number of non-aggregated RBC in hemocytometer. The total number of RBC in a probe counted in the isotonic solution of NaCl. A level aggregation counted by formulae A = 100%—(a number of free (non-aggregated) RBC, x—the total number of RBC − 1 × 100%).

The concentration of malonic dialdehyde (MDA) was defined by the formation of tined trimethine complex with the absorption maximum at 530 nm under the reaction with thiobarbituric acid (n = 8/group/time point). To calculate the concentration of MDA the coefficient of molar extinction E = 1,56 × 10-5 M-1sm-1 [45] was used. Catalase activity was analyzed by the decrease in peroxide in the probe. The measurements were made by spectrophotometry on Shimadzu XRD-700 immediately after the introduction of H_2_O_2_ in the pan with the probe and 20 s after the introduction at the wave length 240 nm [46].

ATP and 2.3-BPG concentration measurement was produced by non-enzymatic method (n = 8/group/time point) [7]. ATP and 2.3-BPG concentration was defined in trichloroacetic acid (TCA) filtrate of hemolyzed RBC. When defining ATP equal volumes of TCA filtrate were mixed with 2H HCl and 2H NaOH. Inorganic phosphorus (Pn) in the composition of which there were Pn separated from ATP after hydrolysis and Pn which had been there before the hydrolysis. To define 2.3-BPG the nucleotides (ATP, ADP, AMP) were removed from trichloroacetic acid filtrate of hemolyzed RBC. It was perfomed by the adsorption on activated carbon with the further centrifugation. In supernatant, inorganic phosphate (Pn) was defined (test tube 1). Another part of TCA filtrate was subjected to the process of ashing, with the addition of 5% solution of Mg nitrate and 0.36 N H_2_SO_4_. Pn was measured in supernatant (test tube 2). Pn was defined in each test tube, registering coloring density on KFK-3 photoelectric photometer (λ 660 nm). Pn concentration was defined by gauge curve, using standard solution KH_2_PO_4_. 2.3-BPG concentration calculation was according to the formulae (Pn (test tube 1) × 100 − Pn (test tube 2) × 10) / 2 [47].

### Histopathology

Histological examination were carried out on the 1st, 3rd, 7th and 12th days with rats suffered TBI after blood sampling. Decapitation animals took place under Na thiopental anesthesia, in a dose 100 mg/kg animal mass. After decapitation (n = 8/group/time point), the brain was quickly removed from each rat, dissected, and was put into 10% buffered neutral formalin solution. Material fixation had been lasting for 72–96 h, then bits of parietal occipital regions of the brain was cut from the fixed material for the following histological investigation. For this purpose they were embedded into paraffin (the media Histomix-extra, “Biovitrum”, Russia were used). The slices 5–7 mkm thick on the rotation microtome Leica 450 RM (Leica Microsystems, Germany) from the received blocks were produced. The slices were colored with hematoxylin and eosin.

A senior pathologist performed an initial histopathological analysis, while blind to the RBCs functional indexes results. He analysis the area (S, mm^2^) of arterioles, venules and capillaries (on cross sections), the area of perivascular edema around the examined vessels (S, mm^2^) and the diameter of capillaries (d, mm^2^) were visualized under a lightmicroscope. In total about 30 brain sections per rat were analyzed. Using 400 × magnification, 20 randomized field were selected randomly and 10 vessels and / or 30 cells were counted in each field. Histological preparations were studied with the light microscope Leica DM1000 (Leica Microsystems, Germany), micro photos were got with the help of digital camera Leica DFC290 (Leica Microsystems, Germany).

### Analysis of animal motion activity

The presence and degree of intensity of motor disorders were defined by using the method of moving on bar [48]. The ability to balance and to stay at the bar (in numbers), time spent for moving on the bar from the bright light source to the darkroom (in seconds), and rats paw sliding frequency (in numbers) were defined in the posttraumatic period.

### Statistical analysis

The received data were processed with the use of the application package BIOSTAT (Analystsoft < USA) and Microsoft Excel (Microsoft, USA) applying the methods of one-dimensional statistics. The results are given as M + SD, where M—arithmetic average, m—standard error of the mean. The authenticity of average differences was defined by Student's *t*-test. The differences were supposed to be reliable when the level of importance was p < 0.05.

## Data Availability

All data generated or analysed during this study are included in this published article.

## Abbreviations

RBC (erythrocyte): Electrophoretic mobility electrophoretic mobility of erythrocytes
LPO: Lipid peroxidation
MDA: Malonic dialdehyde
TBI: Traumatic brain injury
TCA: Trichloroacetic acid
